# Supplementary material for: Achieving ultrahigh triboelectric charge density for efficient energy harvesting
Source: Nat Commun. 2017 Jul 20;8:88. doi: 10.1038/s41467-017-00131-4 (PMC5519710; doi:10.1038/s41467-017-00131-4)
Supplement: Supplementary file 1 — Supplementary Information [file 41467_2017_131_MOESM1_ESM.pdf]

File Name: Supplementary Information
Description: Supplementary Figures and Supplementary Notes

File Name: Supplementary Movie 1
Description: An electronic watch is powered and a capacitor is charged quickly while a TENG works in high vacuum.

File Name: Supplementary Movie 2
Description: An electronic watch is powered and a capacitor is charged slowly while a TENG works in atmosphere.

File Name: Supplementary Movie 3
Description: A temperature-humidity meter is powered and a capacitor is charged while a TENG works in high vacuum.

File Name: Supplementary Movie 4
Description: A temperature-humidity meter is powered and a capacitor is discharged quickly while a TENG works in atmosphere.

File Name: Supplementary Movie 5
Description: Thirty-two LED light bulbs (Each with rated power of 0.75 W) are lit in complete darkness by the TENG working in vacuum.

File Name: Supplementary Movie 6
Description: Two LED light bulbs (Each with rated power of 0.75 W) are lit in complete darkness by the TENG working in atmosphere.

File Name: Peer Review File
Description:

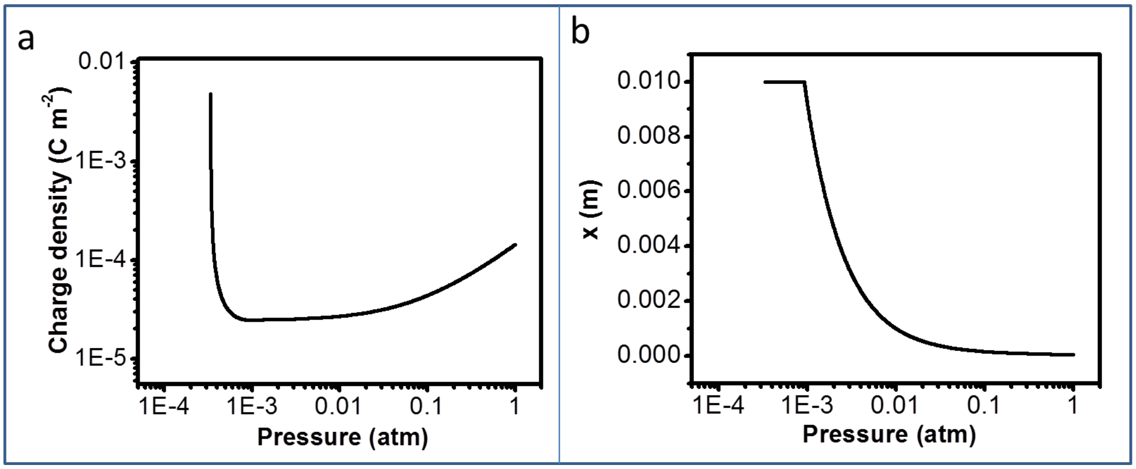

Supplementary Figure 1. (a) The theoretical maximum surface charge density allowed without air breakdown at different air pressures. (b) The corresponding gap distance at which air breakdown is about to occur. The thickness of PTFE film is 200 μm and the maximum gap distance of TENG is 1 cm.

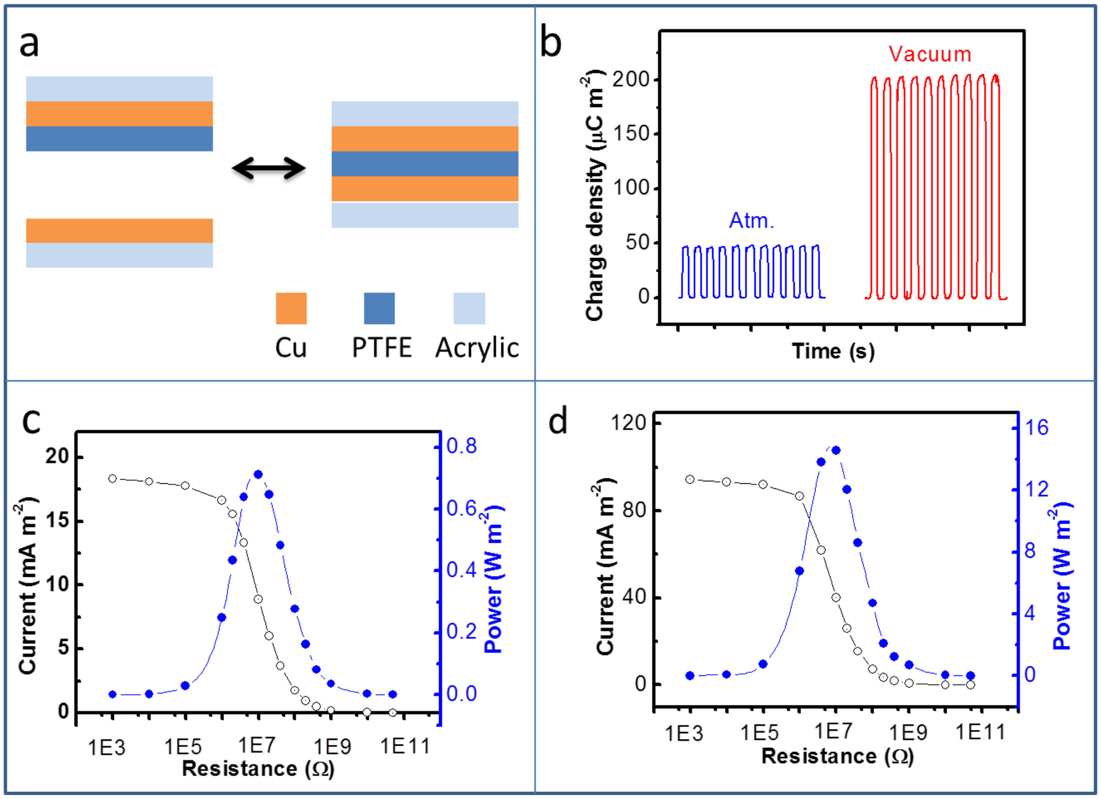

Supplementary Figure 2. Output performance of a TENG based on PTFE/Cu with contact area of 3 cm × 3 cm. (**a**) Schematic of conventional TENG with hard contact. (**b**) Charge density of the TENG in atmosphere and high vacuum ($P \sim 10^{-6}$ torr). (**e-f**) Current density and power density of TENG with various loads in atmosphere (e) and vacuum (f).

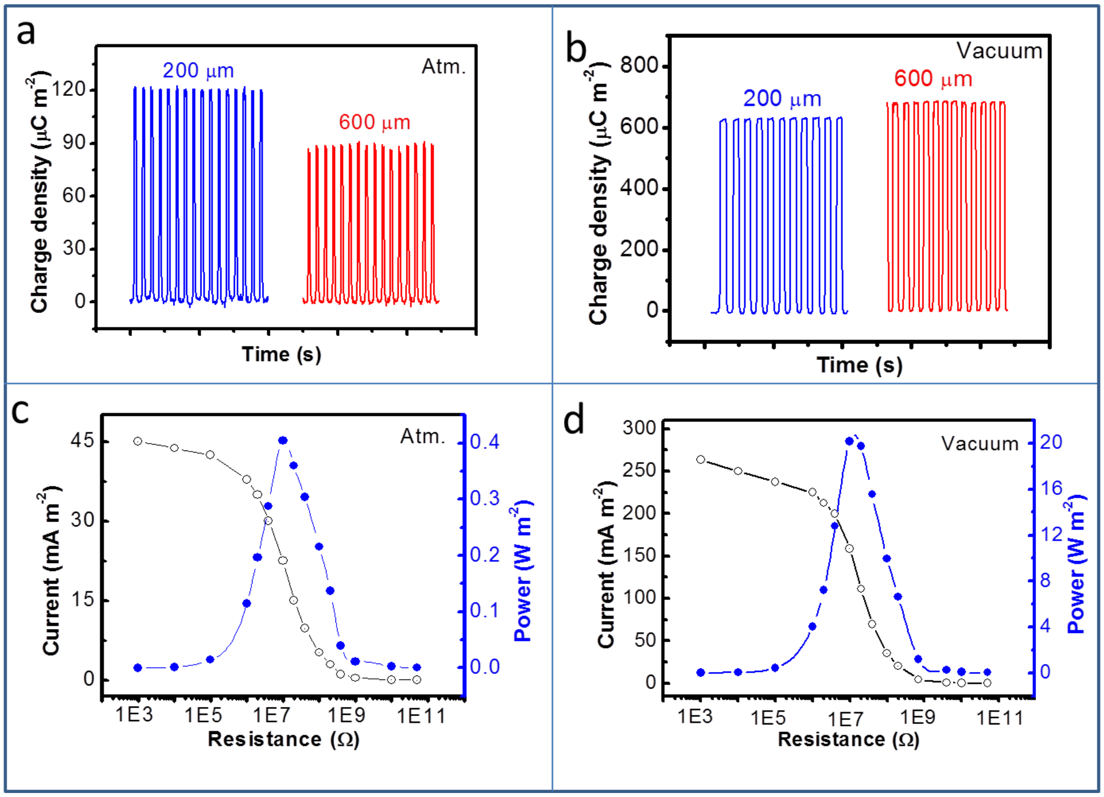

Supplementary Figure 3. Output performance of a TENG with PTFE layer of different thickness in atmosphere and high vacuum. (a-b) Charge density with a 200 μm and a 600 μm thick PTFE layer in atmosphere (a) and vacuum (b). (c-d) Current density and power density of TENG with a 600 μm thick PTFE layer at various loads in atmosphere (c) and vacuum (d).

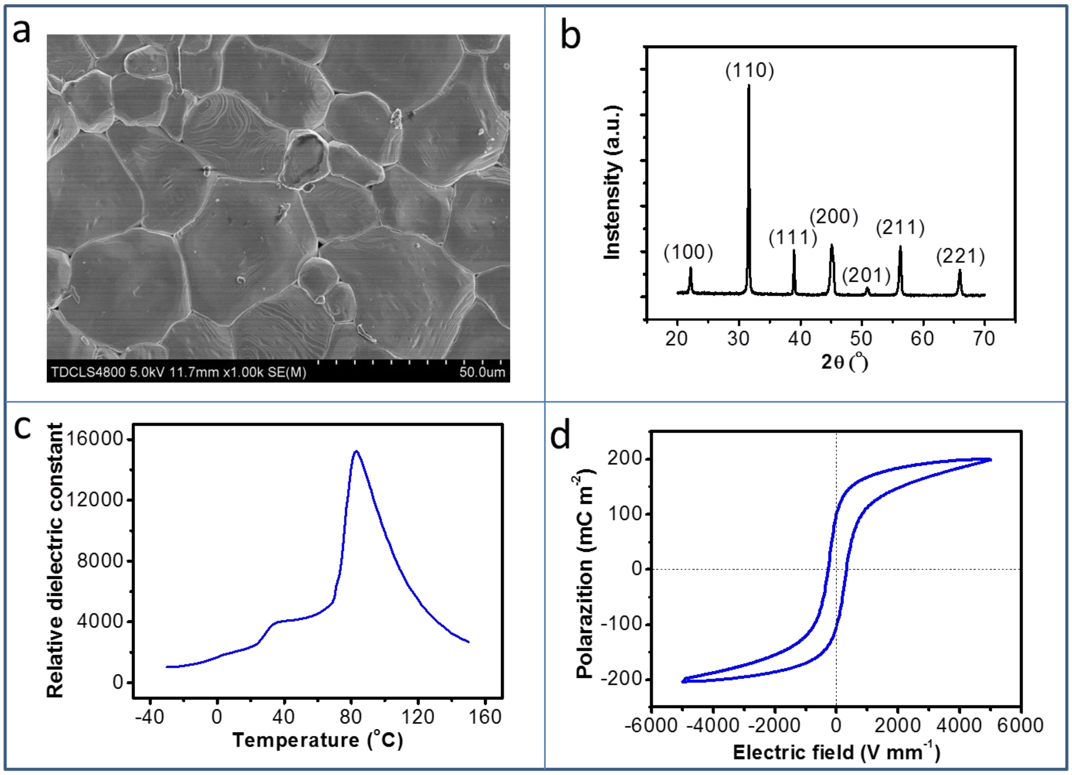

Supplementary Figure 4. Characteristics of BT ceramics. SEM micrograph (a), XRD pattern (b), dielectric permittivity (c) and P-E hysteresis loop (d) for BT ceramics.

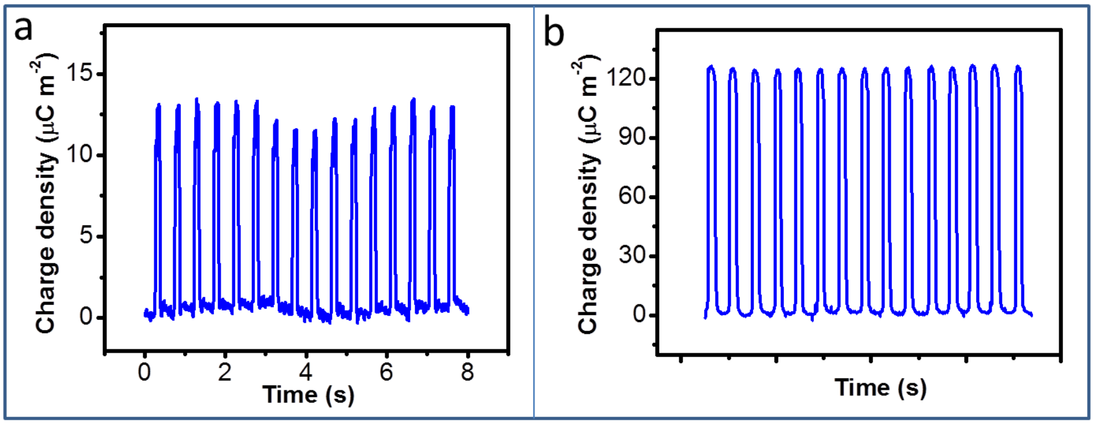

Supplementary Figure 5. Surface charge density of a TENG with BT ceramic (a) and PTFE (b) as the triboelectric layer in atmosphere, where both the BT ceramic and PTFE have a circular diameter of 1 cm.

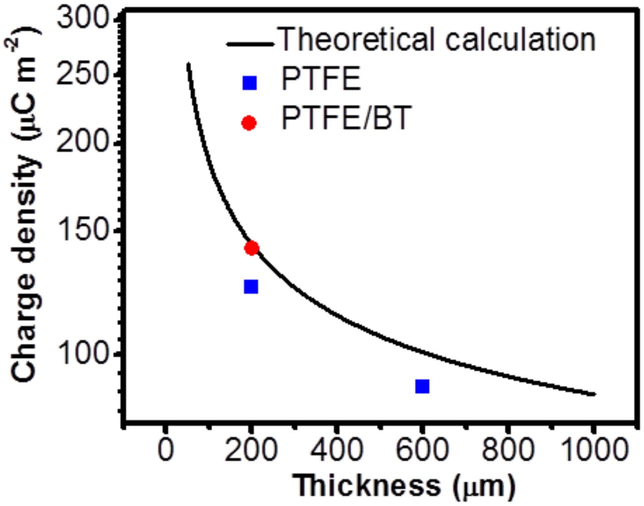

Supplementary Figure 6. Relationship between the maximum surface charge density allowed without air breakdown and the thickness of PTFE film in atmosphere.

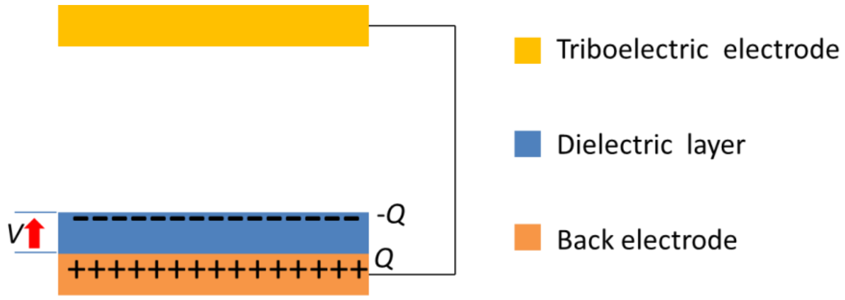

Supplementary Figure 7. Schematic of the electric field in the dielectric layer of TENG, PTFE in our case, when the triboelectric electrode is far enough from the dielectric layer.

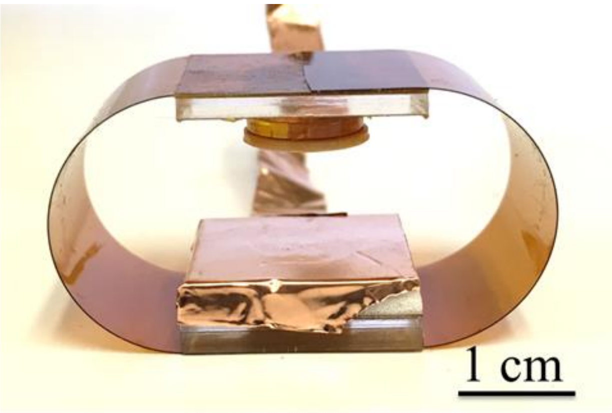

Supplementary Figure 8. Image of the TENG with the coupling of surface and dielectric polarization.

Supplementary Note 1: Theoretical calculation of maximum surface charge density:

As discussed in the main text, the gaseous breakdown voltage can be determined empirically by

$$V_b = \frac{APd}{\ln(Pd) + B} \qquad (1)$$

where A and B are the constants determined by the composition and the pressure of the gas. For air at standard atmospheric pressure (1 atm， i.e. the conventional operation condition of a TENG), A = 2.87 × 10 5 V atm^{-1} m^{-1}, and B = 12.6.

Meanwhile, the gap voltage between contact surfaces of a Cu-PTFE TENG (V_{gap}) under short-circuit condition is given by

$$V_{gap} = \frac{t\sigma d}{\varepsilon_0 (t + d\varepsilon_r)} \qquad (2)$$

where t is the thickness of the PTFE film, σ the triboelectric surface charge density, ε_r the relative permittivity of PTFE, and ε_0 the vacuum permittivity.

To avoid air breakdown, the V_{gap} must be smaller than V_b at any operation gap distance ($0 < d < d_{max}$), i.e.

$$\frac{APd}{\ln(Pd) + B} - \frac{t\sigma d}{\varepsilon_0 (t + d\varepsilon_r)} > 0 \qquad (3)$$

for all $d \in [0, d_{max}]$. Therefore, the maximum surface charge density allowed on a dielectric film of thickness t and relative permittivity ε_r at a given air pressure P, is given by

$$\sigma_{max} = \min\left(\frac{AP\varepsilon_0 (t + d\varepsilon_r)}{t(\ln(Pd) + B)} \right) \qquad (4)$$

Using Equation 4 and with the assumption of constant coefficients A and B, the maximum surface charge density allowed without air breakdown at different pressures is calculated and plotted in Supplementary Fig. 1a. The corresponding gap distance at which the air breakdown is about to occur is also calculated and presented in Supplementary Fig. 1b. It can be observed that the surface charge density leading to air breakdown first decreases

with the decreasing gas pressure and then increases with the further decreasing of the pressure. More than 4000 μC m^{-2} is theoretically allowed when the pressure is about 10^{-4} atm and the gap distance of TENG is less than 1 cm. Using the same equation 4, the relationship between the maximum surface charge density allowed without air breakdown and the thickness of PTFE film in atmosphere can be obtained as well, with results shown in Supplementary Fig. 6.

Supplementary Note 2: Performance of a conventional TENG in atmosphere and high vacuum: For reference, we fabricated a conventional contact-separation TENG by directly using Cu electrodes and a PTFE dielectric layer. The size is 3 cm × 3 cm with no soft foam under the triboelectric Cu electrode (Supplementary Fig. 2a). Its surface charge density is about 46 μC m^{-2} in atmosphere (Supplementary Fig. 2b), which agrees with previously reported values. Its charge density is improved to 203 μC m^{-2} in high vacuum, giving an over 3-fold enhancement. As a result, the output power density is improved from 0.71 W m^{-2} (Supplementary Fig. 2 c) in atmosphere to 14.6 W m^{-2} (Supplementary Fig. 2 d), giving an around 20-fold enhancement.

Supplementary Note 3: Effect of dielectric thickness on the performance of TENG: In atmosphere, the breakdown voltage of TENG is related to the thickness of the dielectric layer. For TENGs with cushioned Cu electrodes, the surface charge density decreases from 120 μC m^{-2} to 90 μC m^{-2} when the thickness of the dielectric PTFE film increases from 600 μm to 200 μm (Supplementary Fig. 3 a). In high vacuum, the TENG with 200 μm-thick PTFE reaches 630 μC m^{-2}, and the TENG with 600 μm measures up to 680 μC m^{-2}. The slightly higher charge density of the latter can be attributed to the greater contact intimacy when PTFE film is thicker. Accordingly, the maximum output power density of the TENG with thicker dielectric film is improved from 0.4 W m^{-2} in atmosphere (Supplementary Fig. 3 b) to 20 W m^{-2} (Supplementary Fig. 3 c) in high vacuum, giving an around 49-fold enhancement. The independence of charge density and thickness of dielectric layer in high vacuum demonstrates that the charge density of contact electrification in atmosphere is limited by air breakdown. Without concern about air breakdown, thick dielectric films can be used in TENGs working in high vacuum, which would bring about better mechanical robustness.

Supplementary Note 4: Characteristics of BT samples: A dense structure with an average grain size of about 22 μm was observed in the fabricated BT samples (Supplementary Fig. 4a). As shown in Supplementary Fig. 4 b, the specimen exhibited a perovskite structure and traces of impurity were detected. The dielectric permittivity of poled BT samples was measured from -30 ℃ to 150 ℃ at 1 kHz (Supplementary Fig. 4 c). Two obvious peaks were detected at about 84 ℃ and 30 ℃, respectively. The relative dielectric constant ε_r at the former peak, corresponding to the ferroelectric-paraelectric phase transition, could reach ~15250. Meanwhile, the ε_r, at the later peak that is corresponds to the transition from rhombohedral to tetragonal phase, could also reach 4000,

indicating that the BT samples possess excellent dielectric properties. The polarization-electric field (P-E) hysteresis loops were measured at 5 kV mm^{-1} (Supplementary Fig. 4 d). It was observed that the BT ceramics exhibit a “slim” P-E loop with small coercive field E_c due to the “soft” nature of BCZT ceramics. Giant remnant polarization (P_r) of ~100 mC m^{-2} and spontaneous polarization Ps of ~200 mC m^{-2} were also detected.

Supplementary Note 5: Calculation of the equivalent galvanostatic current: The equivalent galvanostatic current (I_{eg}) is calculated by equation:

$$I_{eg} = C \times \Delta V / \Delta t \qquad (5)$$

where C is capacitance of the capacitor, ΔV represents the voltage change during the charging/discharging time (Δt).

Supplementary Note 6: Calculation of the $\sigma_{\text{Dielectric breakdown}}$ of dielectric layer: The surface charge density σ is defined as:

$$\sigma = Q/S \qquad (6)$$

where S is the surface area, and Q is the total surface charge quantity. When the triboelectric electrode is far enough from the dielectric layer after contact electrification (Supplementary Fig. 7), all the triboelectric charges on the triboelectric electrode will flow to the back electrode, and the dielectric layer can be regarded as a capacitor with opposite charges of Q on the top and bottom surfaces. Therefore, the charge Q can also be expressed as

$$Q = CV \qquad (7)$$

where C is the capacitance of dielectric layer and can be calculated by Equation 5 where ε_0 and ε_r are the dielectric constant of vacuum (8.85 × 10^{-12} F m^{-1}) and relative dielectric constant of dielectric material respectively. V is the applied voltage on the dielectric layer and it is the product of electric field strength (E) and the thickness (d), as shown in Equation 6.

$$C = \frac{\varepsilon_0 \varepsilon_r S}{d} \qquad (8)$$

$$V = Ed \qquad (9)$$

By combining Equations 6 - 9, we can get Equation 10.

$$\sigma = \varepsilon_0 \varepsilon_r E \qquad (10)$$

Therefore, the surface charge density that will bring dielectric breakdown ($\sigma_{\text{Dielectric breakdown}}$) can be calculated by Equation 8,

$$\sigma_{dielectric\_breakdown} = \varepsilon_0 \varepsilon_r E_{breakdown} \qquad (11)$$

where $E_{\mathrm{breakdown}}$ is the breakdown electric field strength of dielectric materials. For PTFE, ε_r = 2.1 and $E_{\mathrm{breakdown}}$ = 60 MV m^{-1}, then $\sigma_{\mathrm{dielectric\_breakdown}}$ can be calculated to be 1115 μC m^{-2}.

Supplementary Note 7: Preparation of doped BT: Samples of doped BT ceramics were synthesized by the conventional solid-state reaction process, and the preparation process was introduced in detail in our previous work. The raw material, BT without ZnO, was ball-milled in ethanol for 4 h. The dried mixtures were calcined at 1300 ℃ for 2 h, and then mixed by milling with ZnO powder for other 4 h. Mixed powder was pressed into discs with a diameter of 10 mm and consolidated by isostatic pressing at 200 MPa after adding polyvinyl alcohol, as a binder for granulation. Green compacts were sintered at 1450 ℃ for 4 h after burning out the binder at 650 ℃ for 30 min. All samples fabricated had a circular diameter of 1 cm and a thickness of around 0.6 mm.

Supplementary Note 8: Characterization methods of doped BT structure: The phase structure of samples was analyzed by X-ray diffractometer (XRD, Rigaku D/Max 2500, Tokyo, Japan) using Cu Kα1 radiation as the radiation source (λ= 1.5418 Å). Scanning electron microscopy (SEM, S4800, Hitachi, Japan) was used to observe the microstructure of the ceramics. Silver electrodes were painted on the top and bottom surfaces of the polished samples and fired at 600 ℃ for 15 min. Sintered samples were poled in silicone oil at room temperature under 4.0 kV mm^{-1} for 20 min. Dielectric properties were measured by an LCR meter (Agilent E4980A, Santa Clara, CA) from -30 ℃ to 150 ℃ at 1kHz. The polarization-electric field (P-E) hysteresis was determined with a ferroelectric tester (aixACCT TF1000, Aachen, Germany) at a frequency of 1 Hz.
